# Clinical Characteristics of Anti-GABA-B Receptor Encephalitis

**DOI:** 10.3389/fneur.2020.00403

**Published:** 2020-05-21

**Authors:** Fei Zhu, Wei Shan, Ruijuan Lv, Zhimei Li, Qun Wang

**Affiliations:** ^1^Department of Neurology, Beijing Tiantan Hospital, Capital Medical University, Beijing, China; ^2^China National Clinical Research Center for Neurological Diseases, Beijing, China; ^3^Beijing Institute for Brain Disorders, Beijing, China

**Keywords:** anti-GABA-B receptor encephalitis, autoimmune encephalitis, clinical characteristics, positron emission tomography/computed tomography, electroencephalogram, magnetic resonance imaging, seizure

## Abstract

**Objective:** Anti-GABA-B (gamma aminobutyric acid-B) receptor encephalitis is an autoimmune disease mediated by GABA-B-related antibodies. To fully understand the disease, we collected clinical data from patients with GABA-B receptor encephalitis and conducted an analysis to draw conclusions.

**Methods:** All patients with GABA-B receptor encephalitis from the Neurology Department of Beijing Tiantan Hospital, affiliated with Capital Medical University, from August 2015 to September 2019 were accepted as study subjects. The clinical data of the patients were analyzed retrospectively and included the general demographic characteristics, clinical manifestations, and auxiliary examinations, including laboratory results, electroencephalograms (EEGs), brain magnetic resonance imaging (MRI), and positron emission tomography (PET-CT) results, as well as treatments.

**Results:** From August 2015 to September 2019, 14 cases of anti-GABA-B receptor encephalitis were diagnosed. Among these patients, middle-aged and elderly men were the main demographic, with an average age of 52 years; moreover, the onset of the disease was relatively sudden, and the time from onset to diagnosis was ~1 month. The main clinical symptoms were frequent epileptic seizures, cognitive dysfunction, and mental behavioral disorders. In seven (50%) patients, the leukocyte in cerebrospinal fluid (CSF) were increased. Five (36%) patients had elevated cerebrospinal fluid protein. In most patients, the oligoclonal bands (83%) of CSF were positive, and 24 h IgG levels (92%) were increased. Anti-Hu or anti-Yo antibodies were positive in two (14%) patients. Tumor markers in 10 (71%) patients indicated that neuron-specific enolase, gastrin-releasing precursor, non-small cell lung cancer-related antigen, or carcinoembryonic antigen levels were increased. EEG results often indicated slow waves, sharp waves, or spike waves in temporal areas. Brain MRI always showed high T2 signals in the medial temporal lobe, hippocampus, and amygdala and swelling in the medial temporal lobe and hippocampus. PET-CT scans almost showed abnormal metabolism in the hippocampus and temporal lobe. Three (21%) patients who underwent systemic PET-CT showed hypermetabolism in pulmonary parenchymal nodules and enlargement of mediastinal lymph nodes. All patients underwent high-dose hormone therapy or immunoglobulin immunotherapy. After treatment, the symptoms of epilepsy, cognitive disorders, and mental behavioral disorders improved to varying degrees. However, one patient died of lung cancer.

**Conclusion:** Anti-GABA-B receptor encephalitis mainly occurred in middle-aged and elderly men, and the disease onset was relatively sudden. Before disease onset, some patients experienced fever and non-specific respiratory symptoms, which mainly manifested as frequent epileptic seizures, cognitive dysfunction, and abnormal mental behavior. MRI and PET-CT revealed abnormal signals and local metabolism, respectively, in the temporal lobe. Moreover, the disease has a close relationship with lung cancer, which requires long-term follow-up observation.

## Introduction

Autoimmune encephalitis is a disease involving the limbic system and is mainly triggered by infection or autoimmune mechanisms. It can also have a relationship with neuro-antibodies that appear in response to cancer, such as those involved in paraneoplastic syndrome (1–4). In recent years, an increasing number of autoimmune encephalitis-related antibodies have been identified. Lancaster et al. reported anti-GABA-B (gamma aminobutyric acid-B) receptor encephalitis for the first time in 2010 (5). This disease is a type of autoimmune encephalitis mediated by GABA-B receptor antibodies and often involves the limbic system. Clinically, epilepsy, cognitive dysfunction, and mental behavioral abnormalities are the main manifestations, and epilepsy is often the first symptom (6). Recently, there has been an increasing number of reports on anti-GABA-B receptor encephalitis, which expands the understanding of this disease. For example, Ohta et al. reported a case of GABA-B encephalitis with multifocal hyperperfusion in the epithelia and subcortical MRI (7). Kitazaki et al. reported a case of GABA-B encephalitis with syncope (8). Yao et al. summarized seven, eight and 11 patients, respectively (9–11). The results showed that the clinical characteristics of anti-GABA-B encephalitis are intractable epilepsy, mental disorders, and cognitive dysfunction. Multiple antiepileptic drugs are important for the treatment of intractable epilepsy, and the disease is closely related to tumors. Lin et al. studied the risk of death and death-related factors in 28 patients with GABA-B encephalitis. The results showed that the 5-years mortality rate of patients with GABA-B encephalitis was high. Older age at disease onset, the presence of tumors, the number of complications, and deep vein thrombosis (DVT) are associated with death (12). To advance our understanding of this disease, in this study, we collected these cases for further analysis and summary.

## Materials and Methods

In this study, we collected all patients with GABA-B receptor-related encephalitis in the Department of Neurology, Beijing Tiantan Hospital, affiliated with Capital Medical University, from August 2015 to September 2019. All patients were positive for the GABA-B antibody in the blood or cerebrospinal fluid (CSF). We analyzed their clinical characteristics, including general demographic characteristics (age and sex), present history, clinical manifestations (inducement, symptoms), past medical history, auxiliary examination characteristics (laboratory examination, electroencephalogram, electromyography, brain MRI, positron emission tomography), and treatment.

Laboratory tests included routine, biochemical, oligoclonal zone and 24 h IgG CSF tests; serum and CSF autoimmune assays for encephalitis-associated antibodies, such as GABA-B, glutamate decarboxylase 65 (GAD65), N-methyl-D-aspartate receptor (NMDAR), LGI1, contactin-associated protein-2 (CASPR2), α-amino-3-hydroxy-5-methyl-4-isoxazolepropionic acid receptor (AMPAR), antibodies, and paraneoplastic syndrome-associated antibodies, such as Ri, Hu, Yo, amphiphysin, CV2/CRMP5, paraneoplastic antigen MA2 (PNMA2) antibodies; assays for serum and CSF viral antibodies, such as IgG and IgM antibodies to rubella virus, measles virus, toxoplasma, cytomegalovirus, herpes simplex virus, Epstein-Barr (EB) virus, and coxsackie virus; autoantibody spectra including the anti-neutrophil cytoplasmic antibody, anti-nuclear antibody, anti-cardiolipin antibody, rheumatoid factor, anti-streptolysin O, and complement; thyroid function assays including total T3 (TT3), total T4 (TT4), free T3 (FT3), free T4 (FT4), thyroid-stimulating hormone (TSH), thyroglobulin (TG); assays for thyroid-related antibodies included anti-thyroglobulin antibody (TG Ab), anti-thyroperoxidase antibody (TPO Ab), thyrotropin receptor antibody (TRAb); and tests for tumor markers including cytokeratin 19 fragment (CYFRA21-1), alpha-fetoprotein (AFP), carcinoembryonic antigen (CEA), carbohydrate antigen 125 (CA125), carbohydrate antigen 19-9 (CA19-9), carbohydrate antigen 72-4 (CA72-4), carbohydrate antigen 15-3 (CA15-3), neuron-specific enolase (NSE), squamous cell carcinoma antigen (SCC), pro-gastrin-releasing peptide (ProGRP), and β-human chorionic gonadotropin (β-HCG).

Magnetic resonance imaging (MRI), with or without gadolinium injection, was performed using an NT 3.0-T Philips Gyroscan (Eindhoven, the Netherlands). A computed tomography (CT) scan was carried out using a Siemens Somatom Emotion CT machine (Erlangen, Germany). Ultrasonography was performed using a 3.5-MHz convex transducer (Toshiba SSA-250 A or Aloka SSD-630). ^18^F-FDG positron emission tomography (PET) images were acquired using a PET/CT scanner (Elite Discovery, GE HealthCare, Fairfield, Connecticut, USA).

We performed the analysis using SPSS 19.0. Descriptive statistics were used to analyze clinical data, such as the median and percentages.

## Results

### General Demographic Characteristics

Among the 14 patients, nine were male, and five were female. The patients' ages ranged from 23 to 69, and their average age was 52. The onset of the disease was relatively sudden, with an average of 30 days (20 days to 2 years) elapsing from onset to diagnosis. Before the attack, three patients had a fever as high as 38.5°C. Another two patients had non-specific respiratory symptoms.

### Clinical Manifestations

#### Epilepsy

All patients (100%) had epilepsy, with frequent attacks occurring up to four to five times/day. Epilepsy could manifest in two main forms. The first form comprised paroxysmal limb stiffness convulsions, followed by loss of consciousness and limb convulsions, which could be accompanied by head and eye deflections to one direction or both eyes turning upwards, an askew angle of the mouth, spitting, clenched teeth, tongue biting, and urinary incontinence, lasting for several minutes and considered to be secondary tonic-clonic epilepsy. The second form comprised paroxysmal absence seizures, which exhibited similar symptoms, including stupefaction, swallowing, mouth twitching, head numbness, vomiting, and a purple face, and was considered what we called complex partial seizures. A third type comprised simple partial seizures, which manifested through paroxysmal tongue cramps, paroxysmal facial convulsions, or paroxysmal head deflection to one side, with both eyes turned upwards, clear senses, and relief after about 1 min. One patient experienced an initial simple partial attack, followed by a complex partial attack and then a secondary tonic-clonic attack. Two patients experienced an initial complex partial attack and then a secondary tonic-clonic attack. Three patients experienced complex partial attacks and secondary tonic-clonic attacks almost at the same time. Among these individuals, one patient in an epileptic persistent state showed non-convulsive status epilepticus, manifested as slow responses and irrelevant answers; after intravenous injection of diazepam, the symptoms improved.

#### Cognitive Function Impairments

11/14 patients had poor memory, especially related to decreased recent memory abilities and slow responses.

#### Mental Behavioral Abnormalities and Character Change

9/14 patients had mental behavioral abnormalities and character alterations. Mental behavioral abnormalities manifested as non-sensical and forced behaviors. Four patients slept more, whereas two were restless and did not sleep at night. Personality changes manifested as a loss of interest in things, a worsened temper, increased aggressive behaviors such as beating and scolding people, high emotional instability, and an increased tendency to become sad.

#### Other Clinical Characters

One patient walked unsteadily in the initial stage and another patient had difficulty understanding and expressing, she could only understand simple questions but replied with irrelevant answers (Table 1).

**Table 1:** Demographics and clinical characteristics.

**Basic information**			**Main symptoms**	**Medical history**
**Patients**	**Sex**	**Onset**		
1	Male	52	Fever before disease onset, a maximum temperature of 38.5°C, with chills, night sweats, headache, and dizziness; GTCS, decreased memory, mental behavioral abnormalities, somnolence, and unstable walking	Long-term smoking and drinking history
2	Male	44	CPS, decreased memory	Normal
3	Male	62	CPS, GTCS, decreased memory, mental behavioral abnormalities, poor sleep	Hypertension, diabetes, coronary heart disease, tuberculous pleurisy
4	Female	50	CPS, GTCS, decreased memory	Hypertension
5	Female	23	CPS	Normal
6	Female	30	Fever before disease onset, maximum temperature of 38°C with headache; CPS, GTCS, decreased memory; hearing comprehension and expression disorders	Normal
7	Male	50	GTCS, decreased memory, mental behavioral abnormalities	Normal
8	Female	69	GTCS, decreased memory, mental behavioral abnormalities	Rheumatoid arthritis
9	Male	54	CPS, GTCS, mental behavioral abnormalities	Hypertension, hepatitis B
10	Male	69	CPS, GTCS, decreased memory, mental behavioral abnormalities	3-years history of brain injury, hypertension, pulmonary fibrosis, cerebral infarction, bilateral carotid plaque formation, atrial fibrillation
11	Male	52	Cold before onset, GTCS, decreased memory	Chronic nephritis, brucellosis
12	Male	45	Cold before onset, GTCS, CPS, SPS, decreased memory, mental behavioral abnormalities	Normal
13	Male	69	Fever before onset, maximum temperature of 38.1°C, GTCS, mental behavioral abnormalities	Lumbar disc protrusion, atrial fibrillation
14	Female	67	GTCS, non-convulsive status epilepticus, mental behavioral abnormalities, somnolence	Normal

### Auxiliary Inspection

#### Laboratory Inspection

The lumbar puncture pressure was increased in 3/13 patients but was normal in other patients. In 7/14 patients, the leukocytes were increased in CSF. Furthermore, CSF protein was increased in 5/14 patients. All patients had normal CSF glucose and chloride levels. In 10/12 patients, the CSF oligoclonal zone (OB) was positive, and in 12/13 patients, the 24 h IgG level in CSF was increased.

Twelve patients were positive for GABA-B antibody in CSF and blood, and only two patients were positive for the GABA-B antibody in CSF. Other autoimmune encephalitis-related antibodies, including other neuron-specific antibodies in the blood and CSF (such as NMDA, CASPR2, AMPA1, AMPA2, LGI1, and GAD65 antibodies related to autoimmune encephalitis and Ri, Hu, Yo, amphiphysin, CV2/CRMP5, and PNMA2 antibodies related to paraneoplastic syndrome), were negative in 12 patients. Two patients were positive for paraneoplastic antibodies, one patient was slightly positive for anti-Yo antibody in blood, and one patient was positive for CSF and anti-Hu antibodies in blood and CSF.

Among the 14 patients, there were four with normal tumor markers, and the remaining 10 patients had different types of tumor markers that were increased to varying degrees, including six patients with increased NSE levels, four patients with increased serum gastrin-releasing peptide precursor (ProGRP) levels, three patients with increased non-small cell lung cancer-associated antigen (CYFRA21-1) levels, three patients with carcinoembryonic antigen (CEA) levels, one patient with increased CA19-9 levels, one patient with increased CA242 levels, one patient with increased CA72-4 levels, one patient with increased total prostate-specific antigen (t-PSA) levels, and one patient with elevated squamous cell carcinoma-associated antigen levels.

All (13/13) patients' IgM antibodies to virus (rubella virus, measles virus, toxoplasma, cytomegalovirus, herpes simplex virus, EB virus and coxsackie virus) either in CSF or in blood were negative. Cytomegalovirus IgG antibody was positive in CSF of 11/13 patients. All (13/13) patients had positive IgG antibodies to cytomegalovirus, herpes simplex virus, and EB virus in blood. Ten out of 13 patients were positive for rubella virus IgG antibody in blood.

All patients' autoantibody spectra were examined, including anti-neutrophil cytoplasmic antibody, anti-nuclear antibody, anti-cardiolipin antibody, rheumatoid factor, anti-streptolysin O, and complement. One patient was slightly positive for Scl-70, three were positive for Ro-52, one was positive for histone, and one was positive for gastric parietal cells. All patients were negative for rheumatoid factor, anti-O antibody and anti-neutrophil cytoplasmic antibody. All patients were examined for thyroid function: three patients were abnormal, and 11 were normal (Tables 2, 3).

**Table 2:** CSF profiles.

**Patients**	**CSF**				
	**Pressure**	**WBC/total cells/ul**	**Biochemistry**	**Oligoclonal zone**	**24 h IgG**
1	N/A	41/141, Monocyte 48.7%;	Normal	Positive	↑
2	120	5/205, N/A	Normal	Positive	↑
3	145	20/20, N/A	P:53.72 mg/dL	Positive	↑
4	150	2/2, N/A	Normal	Negative	Normal
5	170	7/7, Monocyte 14.3%;	Normal	Positive	↑
6	280	368/468, Monocyte 48.1%	Normal	Negative	↑
7	140	2/2, N/A	Normal	Positive	↑
8	145	3/103, Monocyte 33.4%	Normal	Positive	↑
9	230	15/1415, Monocyte 40%	P:69.29 mg/dL	N/A	↑
10	120	35/735, Monocyte 11.4%	Normal	Positive	↑
11	90	16/416, N/A	Normal	Positive	↑
12	230	8/8, N/A	P:77.46 mg/dL	Positive	↑
13	105	6/6, N/A	P:46.03 mg/dL	N/A	N/A
14	105	19/19, Monocyte 11%	P:63.27 mg/dL	Positive	↑

*↑, increased*.

**Table 3:** Serological profiles.

**Patients**	**Lab Tests**				
	**AE Antibodies**	**Virus antibody(IgG)**	**Virus antibody(IgM)**	**Tumor Markers**	**Thyroid function, Thyroid-associated antibodies, Autoimmune antibody**
1	GABAB antibody: 1:320 (Blood), 1:100 (CSF)	Cytomegalovirus antibody (CSF) herpes simplex virus, cytomegalovirus, rubella virus, EB virus (Blood)	Negative	NSE↑, PROGrp↑	Normal Scl-70 antibody↑
2	GABAB antibody: 1:320 (Blood), 1:320 (CSF)	Cytomegalovirus antibody (CSF) herpes simplex virus, cytomegalovirus, rubella virus, EB virus (Blood)	Negative	NSE↑	Normal
3	GABAB antibody: 1:320 (Blood), 1:320 (CSF)	Cytomegalovirus antibody (CSF) herpes simplex virus, cytomegalovirus, rubella virus, EB virus (Blood)	Negative	ROGrp↑, CYFRA21-1↑, NSE↑	Normal Ro-52 antibody↑
4	GABAB antibody: 1:320 (Blood), 1:320 (CSF)	Cytomegalovirus antibody (CSF) herpes simplex virus, cytomegalovirus, rubella virus, EB virus (Blood)	Negative	CA72-4↑	Normal
5	GABAB antibody: 1:100 (Blood), 1:100 (CSF)	Cytomegalovirus antibody (CSF) herpes simplex virus, cytomegalovirus, rubella virus, EB virus (Blood)	Negative	Normal	Normal
6	GABAB antibody: 1:10 (Blood), 1:10 (CSF), Yo-antibody: positive (Blood)	Cytomegalovirus antibody (CSF) Herpes simplex virus, cytomegalovirus, rubella virus, EB virus (Blood)	Negative	CA19-9↑, NSE↑	TT3↓FT3↓ Ro-52 antibody↑
7	GABAB antibody: Positive (Blood), positive (CSF)	Cytomegalovirus antibody (CSF) Herpes simplex virus, cytomegalovirus, rubella virus, EB virus (Blood)	Negative	PROGrP↑, SCC↑, NSE↑	Normal Antihistone antibody↑
8	GABAB antibody: Positive (Blood), positive (CSF)	Cytomegalovirus antibody (CSF) Herpes simplex virus, cytomegalovirus, rubella virus, EB virus (Blood)	Negative	CEA↑	Normal
9	GABAB antibody: 1:10 (Blood), 1:10 (CSF)	Cytomegalovirus antibody (CSF) Herpes simplex virus, cytomegalovirus, rubella virus, EB virus (Blood)	Negative	CEA↑	TSH↓ Ro-52 antibody↑
10	GABAB antibody: positive (CSF)	Cytomegalovirus, EB virus, Herpes simplex virus (Blood)	Negative	CEA↑, CA242↑, t-PSA↑, CYFRA21-1↑	Normal
11	GABAB antibody: Positive (Blood), positive (CSF)	Herpes simplex virus, cytomegalovirus, EB virus(Blood)	Negative	Normal	Normal
12	GABAB antibody: Positive (CSF)	Cytomegalovirus antibody (CSF) Herpes simplex virus, cytomegalovirus, rubella virus, EB virus (Blood)	Negative	Normal	Normal
13	GABAB antibody: Positive (Blood), positive (CSF)	N/A	Negative	Normal	Normal
14	GABAB antibody: 1:320 (Blood), 1:100 (CSF) Hu-antibody: positive (Blood), positive (CSF)	Cytomegalovirus antibody (CSF) Herpes simplex virus, cytomegalovirus, EB virus (Blood)	Negative	NSE↑, PROGrp↑, CYFRA21-1↑	FT4↑, TG↑ TPO-Ab↑ Anti-parietal cell antibody↑

*↑, increased*.

#### EEG Examination

**Figure 1:**
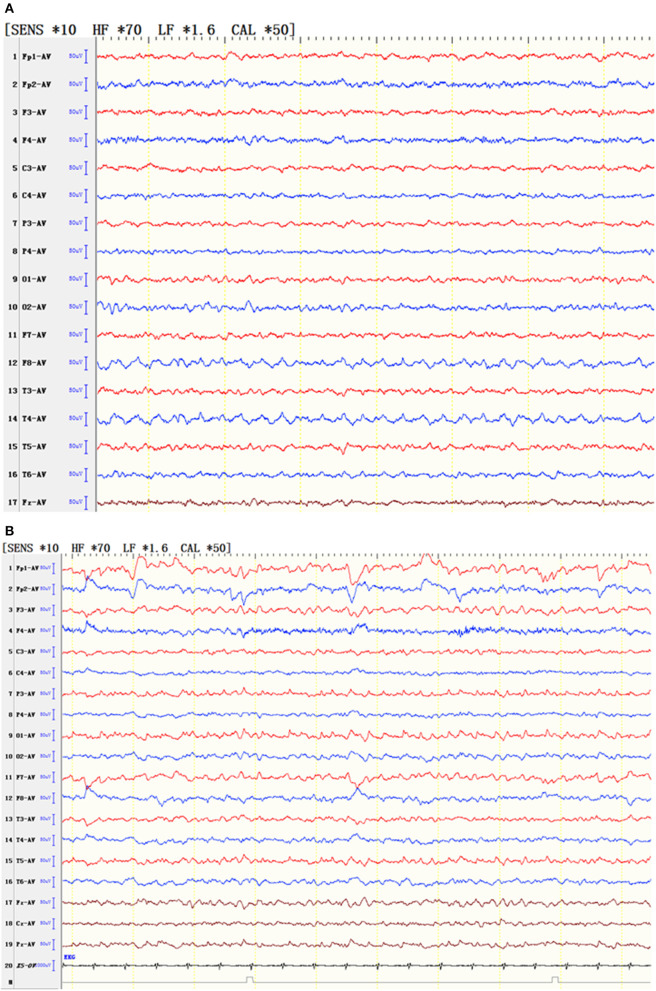
EEG of typical patients. **(A)** EEG showed that the slow wave of the low middle amplitude was seen continuously in the right anterior middle temporal area. **(B)** EEG showed slow waves in the bilateral frontotemporal area and spinous waves in the left posterior parietooccipital temporal area.

Nine patients had sharp waves, spike waves, or slow waves in the temporal area, and six patients had sharp waves or slow waves in the frontal area in conjunction with abnormal waves in the temporal area (Figure 1; Table 4).

**Table 4:** Neuroimaging and neurophysiological features.

**Patients**	**Laboratory tests**		
	**MRI**	**AEEG**	**PET-CT**
1	Bilateral centrum semiovale and lateral paraventricular ischemic white matter lesions	Normal	Head PET-CT: Metabolism of cerebral cortex (except deep occipital cortex) was decreased. Whole body PET-CT: There was no tumor-like hypermetabolism in the whole body
2	Bilateral MTL, abnormal signal	Right temporal lobe sharp wave	PET-CT of brain and trunk showed no obvious tumor such as hypermetabolism.
3	Normal	Interval EEG: left parietal, occipital and posterior temporal lobe slow wave and spike wave, left anterior temporal lobe sharp wave. Episodic EEG: probably originated from the left temporal lobe	The metabolism of left hippocampus, left temporal lobe, bilateral basal ganglia and left upper lobe of lung were increased, considered to be lung cancer. Left hilar and mediastinal lymph node metastasis.
4	Atrophy of right hippocampus	Right frontal lobe and anterior temporal lobe slow waves	Head PET-CT: The metabolism of the left posterior central gyrus was decreased, and suspected epileptogenic focus was considered. Whole body PET-CT: There was no tumor-like hypermetabolism in the whole body
5	Normal	Normal	
6	Multiple patchy and slightly long T1 and slightly long T2 signal shadows in the bilateral frontal lobe, temporal lobe and insula. FLAIR sequence showed strong signal, DWI showed strong signal, and enhanced scanning showed linear enhancement in bilateral frontal temporal pia mater	1.5–2.5 Hz δ wave in bilateral frontal, anterior middle temporal lobe and frontal midline (Fz) areas	
7	A few lacunar and ischemic foci in bilateral corona radiata	Interval EEG: 4–6 Hz θ rhythm in bilateral frontal and midline (Fz) areas, spike wave in the left parietal, occipital and posterior temporal lobes, sharp wave in the left frontal and anterior temporal lobe; Episodic EEG: probably originated from the left anterior temporal lobe	Head PET-CT: The metabolism of the left angular gyrus, the left superior marginal gyrus and the left temporal lobe was decreased, the metabolism of the left hippocampus was increased. Whole body PET-CT: There were small lymph nodes with increased metabolism in the left hilum. It was probably a non-specific inflammatory change.
8	Atrophy of bilateral hippocampus	Interval EEG: sharp and slow waves in the right anterior middle temporal lobe. Left parietal, occipital and posterior temporal sharp waves. Left anterior middle temporal lobe sharp wave. Episodic EEG: probably originated from the right anterior temporal lobe	Head PET-CT: Metabolism of the medial and basal ganglia of both temporal lobes was increased. Whole body PET-CT: Inflammatory lymph nodes of the right hilum; bullae of the upper lobe and fibrous cord shadow of the lower lobe of the right lung
9	Encephalomalacia and chronic hemorrhagic foci in left temporal lobe	Normal	
10	Multiple ischemic white matter lesions	4–6 Hz θ wave and 2.5–3.5 Hz δ wave in the left frontal and anterior middle temporal lobes	Head PET-CT: The metabolism of the medial temporal lobe, amygdala and hippocampus increased, which was consistent with the change in tumor associated encephalitis. Whole body PET-CT: The nodular shadow of metabolic increases in the left lower lobe of the lung was considered to be a malignant disease, with the possibility of lymph node metastasis in the left hilum.
11	Left hippocampal volume slightly reduced	The spike wave and spike slow wave of bilateral frontal pole, forehead, center and anterior middle temporal lobe were not distributed synchronously, and the anterior middle temporal area was obvious, especially the right side	Head PET-CT: The metabolic distribution of bilateral temporal lobe was slightly uneven. Whole body PET-CT: Multiple slightly large lymph nodes in the mediastinum, tending to inflammatory hyperplasia.
12	Gliosis of right hippocampus	Normal	
13	Bilateral MTL and hippocampus, abnormal signal	All leads fast wave	
14	Multiple ischemic white matter lesions	The state of non-convulsive epilepsy was persistent, and the interval EEG was slow and fast in bilateral frontal and anterior middle temporal areas. Attack period: the patient's responses were slow; her answers were not in line with what we asked; and the EEG showed slow wave, sharp wave and fast rhythm in bilateral frontal and anterior middle temporal areas. After an intravenous injection of diazepam, the patient's consciousness gradually cleared, and EEG slow wave signals gradually disappeared at the same time.	Head PET-CT: Bilateral lenticular nucleus and bilateral hippocampal metabolism increased. Whole body PET-CT: There were multiple hypermetabolic lymph nodes in the right hilum, mediastinum and supraclavicular fossa, considered to be lymph node metastasis.

**Figure 2:**
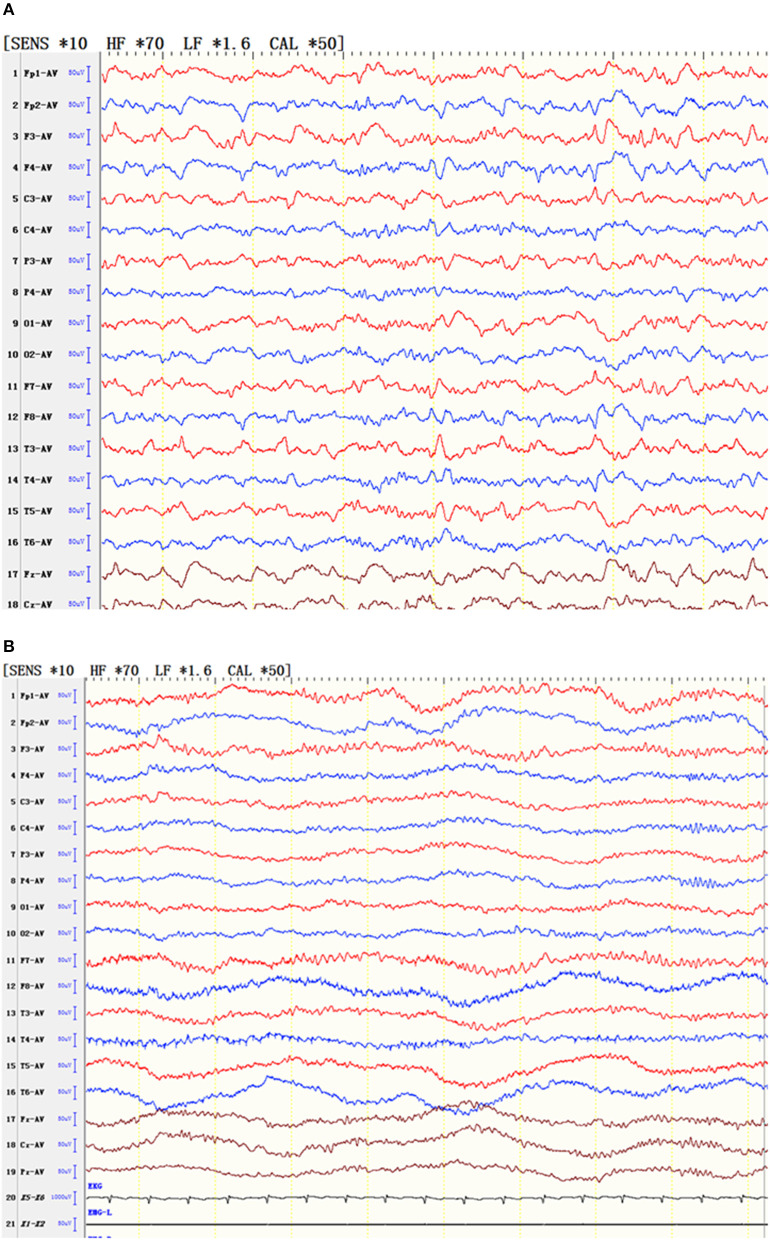
EEG of a patient with status epilepticus. **(A)** During the attack, the patient's responses slowed, and the patient gave irrelevant answers. At the same time, the EEG results showed slow waves, sharp waves and fast rhythms in the bilateral frontal and anterior middle temporal areas. **(B)** After an intravenous injection of diazepam, the patient's consciousness gradually cleared, and the EEG slow wave gradually simultaneously disappeared.

In one patient, the state of non-convulsive epilepsy was persistent, and the interval EEG was slow and fast in the bilateral frontal and anterior middle temporal areas, respectively. In the attack period, the patient's response was slow, the answer she provided did not correspond to what we asked, and the EEG showed slow wave, sharp wave, and fast rhythms in the bilateral frontal and anterior middle temporal areas. After an intravenous injection of diazepam, the patient's consciousness gradually became clear, and the EEG slow wave gradually disappeared at the same time (Figure 2).

### Imaging Examination

#### Brain MRI

**Figure 3:**
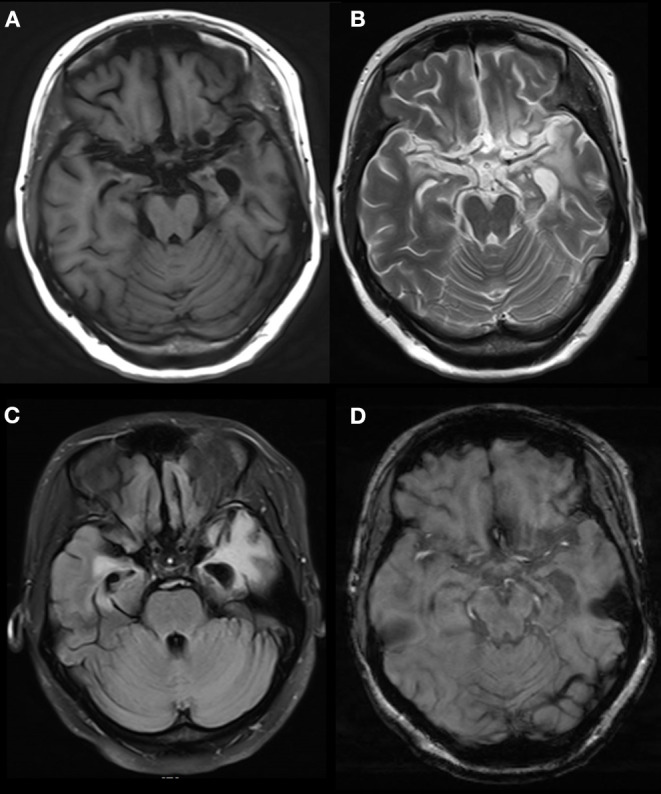
Abnormal signals in bilateral temporal, insular and hippocampal regions indicated by brain MRI. MRI showed a long T2 signal in bilateral temporal, insular and hippocampal slices, with a fuzzy border and uneven signals on the left side. A FLAIR scan showed a strong signal, and no obvious abnormal enhancement was found after injection. The temporal angle of the left lateral ventricle was enlarged, and the midline remained in the middle. MRI, magnetic resonance imaging. **(A)** T1 (T1-weighted imaging). **(B)** T2 (T2-weighted imaging). **(C)** FLAIR (fluid-attenuated inversion recovery). **(D)** T1C (MRI enhanced scan).

**Figure 4:**
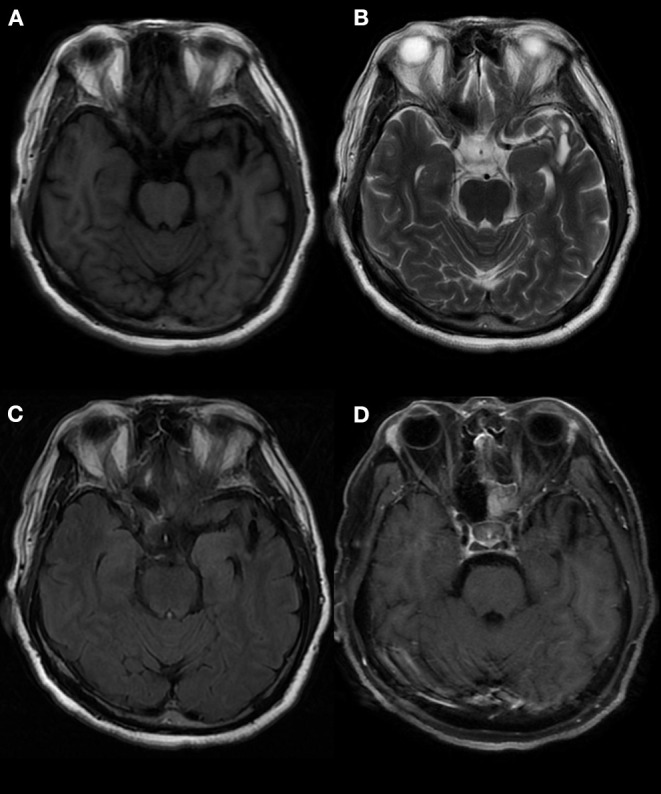
Abnormal signals in the left temporal lobe, as indicated by brain MRI. MRI showed an oval mixed signal shadow in the left temporal cortex, a low signal in the FLAIR scan, a wide left fissure cistern, and no enhancement in the contrast-enhanced scan. MRI: magnetic resonance imaging. **(A)** T1 (T1-weighted imaging). **(B)** T2 (T2-weighted imaging). **(C)** FLAIR (fluid-attenuated inversion recovery). **(D)** T1C (MRI enhanced scan).

In one patient, the MRI showed a long T2 signal shadow in the bilateral temporal, insular, and hippocampal slices, with the left side focus, fuzzy boundary, and uneven signals. The fluid-attenuated inversion recovery (FLAIR) scan showed a strong signal; the diffusion-weighted imaging (DWI) scan showed no diffusion limitation, and no obvious abnormal enhancement shadow was observed after injection. The temporal angle of the left lateral ventricle was enlarged (Figure 3). Seven patients showed abnormal signals in the temporal lobe and hippocampus (Figure 4). There were no specific MRI findings in six patients (Table 4).

#### PET-CT

**Figure 5:**
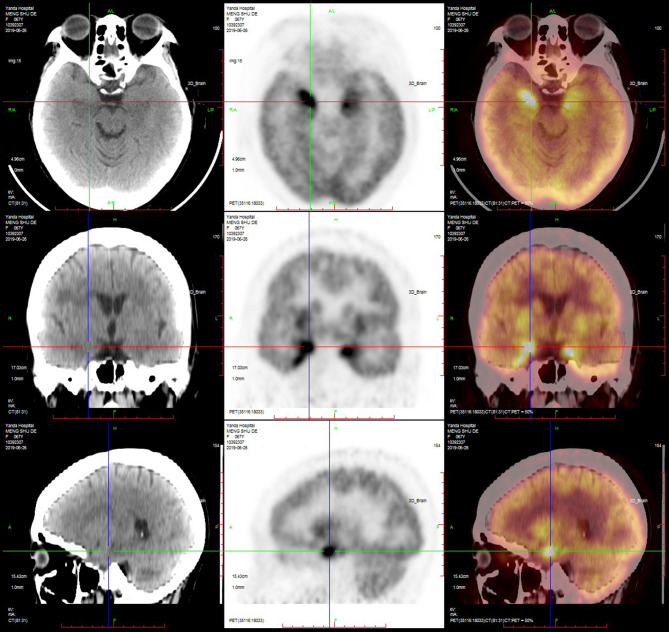
PET-CT of a typical patient. PET-CT suggested that the metabolism of the bilateral medial temporal lobe and hippocampus was increased, especially on the right side.

**Figure 6:**
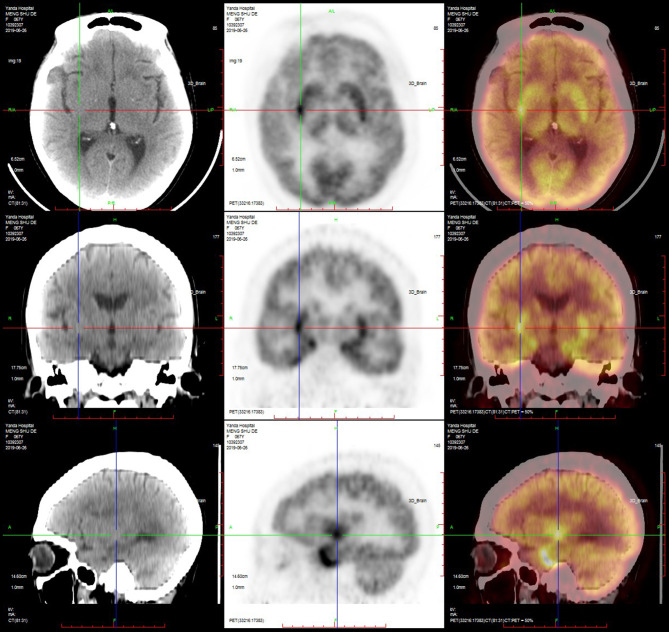
PET-CT of a typical patient. PET-CT suggested that the metabolism of the bilateral basal ganglia was increased, especially on the right side.

PET-CT was performed in nine patients, and the results showed that the metabolism in the hippocampus, temporal lobe, and basal ganglia was increased in six (67%) patients (Figures 5, 6; Table 4).

### Cancer Screening

Tumor screening included abdominal ultrasound, thyroid ultrasound, breast and accessory ultrasound, urinary ultrasound, chest and abdominal computed tomography (CT), and whole-body PET-CT scans. In three patients, lung cancer was found by PET-CT (Table 4). Among them, one patient underwent bronchoscope lavage, and bronchial mucosa biopsy and pathology, which confirmed small cell lung cancer. Another patient underwent a lung biopsy and was pathologically diagnosed with small cell lung cancer too. But the other one refused a pathological biopsy.

### Treatment and Follow-Up Study

Five patients underwent hormone shock therapy, three patients received gamma globulin therapy, and six patients received hormone shock and gamma globulin therapy. All patients were given antiepileptic medicines at the same time. After treatment, the number of epileptic seizures decreased, and mental and behavioral disorders and cognitive decline improved to varying degrees. After treatment, five patients were reexamined for GABA-B antibodies in blood and CSF. The titers of three patients' antibodies decreased, and those of two patients' antibodies became negative. Among the three patients with lung cancer indicated by PET-CT, two patients received radiotherapy and chemotherapy, one patient's tumor disappeared after treatment, however, one patient died. Another lung cancer patient refused radiotherapy or chemotherapy.

## Discussion

Autoimmune encephalitis (AE) generally refers to a kind of encephalitis mediated by autoimmune mechanisms that usually involve the limbic system. The main clinical manifestations are epilepsy, cognitive dysfunction, and abnormal mental behaviors (1, 2).

Since the discovery of anti-NMDAR encephalitis in 2007 (13), a series of autoantibodies against neuronal cell surfaces and synaptic proteins have been found, such as LGI1, GABAR, AMPAR, and CASPR2 antibodies (3, 14, 15). Anti-GABA-B receptor-related encephalitis is a type of rare AE that produces an immune response due to the production of GABA-B receptor-related antibodies.

There is evidence that disruption of the GABA-B-R structure and function can cause spontaneous seizures. Antibodies in the CSF of patients with anti-GABA-B receptor encephalitis can prevent the activation of the GABA-B receptor and block its function without changing the surface density of the receptor *in vitro* (6, 16). Anti-GABA-B receptor encephalitis mainly involves the limbic system and manifests as epilepsy, abnormal mental behavior and memory loss. The disease has a close relationship with small cell lung cancer, for which immunosuppressive therapy is effective, and rapid recognition of the disease is vital because it can improve the outcome of the tumor and nervous system (17).

Anti-GABA-B receptor encephalitis is rare, and the number of reported cases is low. To improve the understanding of this disease, all cases of anti-GABA-B receptor encephalitis in our department were collected, and the clinical data were summarized. According to the data from this group, anti-GABA-B receptor-related encephalitis mainly occurred in middle-aged and elderly men, and sometimes exhibited acute onset, fever, cold and other symptoms before the disease onset, which itself mainly manifested as epilepsy, cognitive function decline (especially recent memory decline), mental behavioral abnormalities and character changes, normal or increased lumbar puncture pressure. Almost half of the patients have elevated cerebrospinal fluid leukocytes. CSF protein can be normal or slightly elevated, and glucose and chloride levels are usually normal.

Patients' blood and CSF OB tests were often positive, and CSF 24 h IgG levels were increased. Blood tumor marker levels were often increased and cytosolic Hu or Yo antibodies in blood and CSF neurons could be positive. The MRI results of patients often indicated abnormal signals in the temporal lobe and hippocampus, and the PET-CT scan of the head often indicated abnormal metabolism of the temporal lobe and hippocampus. Three patients who received systemic PET-CT scans had malignant tumors, and two patients was pathologically diagnosed with small cell lung cancer.

After treatment, the number of epileptic seizures was reduced, and mental behavior and cognitive function were improved to varying degrees. After treatment, blood and cerebrospinal fluid were reexamined in five patients, shown that the titer of GABA-B antibody decreased or became negative.

A prominent feature of anti-GABA-B receptor encephalitis is a relatively rapid onset, with a fever that can occur before disease onset and in some cases, with non-specific respiratory symptoms (18). In our case, three people had fever as high as 38.5°C before the attack, and two people had non-specific respiratory symptoms. For patients with fever and non-specific respiratory symptoms, viral encephalitis caused by viral infection is not excluded. One of our patients had acute onset, fever before the onset of the disease, and a high body temperature of 38°C. This patient's symptoms included headache, epilepsy, slow response, and disordered hearing comprehension and language expression; in addition, this patient's CSF pressure was 280 mm H_2_O, the CSF white blood cells were increased, the CSF OB and 24 h IgG levels were increased too, and CSF ultrawide spectrum pathogenic microorganism detection revealed a low concentration of Mycoplasma pneumonia and human herpes simplex virus type 1. Brain MRI suggested that there were multiple patchy and slightly long T1 and slightly long T2 signal shadows in the bilateral frontal lobe, temporal lobe and insula. The FLAIR sequence showed a strong signal, DWI showed a high signal, and enhanced scanning showed linear enhancement in the bilateral frontal temporal pia mater. Considering that the patient suffered from viral encephalitis, antiviral treatment with acyclovir was given. The total number of cells and leukocytes decreased after lumbar puncture, but the CSF pressure remained high. In addition, GABA-B antibody and Yo antibody in blood and CSF were positive, but symptoms improved after gamma globulin and hormone shock treatment. Therefore, anti-GABA-B receptor encephalitis may have been secondary to viral infection, which should be classified as non-paraneoplastic-related anti-GABA-B receptor encephalitis (17).

In this group, all patients with GABA-B receptor encephalitis experienced epileptic episodes frequently, as many as 4–5 times/day, or even exhibited epileptic status. None of the patients achieved seizure-free status with antiepileptic drugs. One patient with a persistent epileptic state showed non-convulsive status epilepticus, manifested as delayed verbal response and incoherent speech. EEG showed that the fast rhythm in the bilateral frontal and anterior middle temporal areas changed: the amplitude gradually increased, the frequency gradually slowed, and the signal gradually spread to the next to the lead. After an intravenous injection of diazepam, the rhythm improved, indicating non-convulsive status epilepticus, and the symptoms improved significantly after hormone shock and gamma globulin treatment.

It is not surprising that patients with anti-GABA-B receptor encephalitis can show cerebellar ataxia because the GABA-B receptor is highly expressed in the cerebellum (17). Those with the disease can also show language comprehension and expression disorders, similar to those seen in NMDA receptor encephalitis, which may be related to the involvement of the temporal neocortex (19).

Anti-GABA-B receptor encephalitis can be accompanied by other autoantibodies, each with different clinical significance. These can include autoantibodies against Hu, NMDAR, voltage-gated calcium channel (VGCC), glutamic acid decarboxylase 65 (GAD65), and sex-determining region Y-box 1 (Sox1) as well as amphoteric antibodies (6, 11). In our case, one patient was positive for both CSF and serum anti-Hu antibodies, and the patients positive for anti-Hu antibodies had combined small cell lung cancer; thus, the presence of both GABA-B antibodies and Hu antibodies often points to potential lung cancer, which is similar to the results of previous reports (4, 17). Here, one patient was weakly positive for anti-Yo antibody, which is often related to female adnexa, breast and other tumors (20), but this patient had no tumor detected by adnexa or breast examination. There are few reports of anti-GABA-B receptor encephalitis combined with Yo antibody positivity, so we should continue to observe and expand the understanding of this disease in future research.

## Conclusion

Anti-GABA-B receptor encephalitis mainly occurs in middle-aged and elderly men, and the onset of the disease is usually rapid. The main manifestations are frequent epileptic seizures, cognitive dysfunction, and abnormal mental behavior. The focus mainly involves the limbic system. Moreover, the disease is closely related to lung cancer, and long-term follow-up is needed. A comprehensive CSF test is of substantial significance for the treatment of new epileptic cases. A comprehensive understanding of the disease is a good way to prevent misdiagnosis and delayed treatment.

## Data Availability Statement

All datasets generated for this study are included in the article/supplementary material.

## Ethics Statement

The studies involving human participants were reviewed and approved by Beijing Tiantan Hospital Ethics Committee. The patients/participants provided their written informed consent to participate in this study. Written informed consent was obtained from the individual(s) for the publication of any potentially identifiable images or data included in this article.

## Author Contributions

FZ collected and analyzed the data and wrote this article. WS made a substantial contribution to the writing of the article and the production of the chart. RL and ZL collected some data. QW had full access to the data and takes responsibility for the integrity of the data and the accuracy of the analysis.

## Conflict of Interest

The authors declare that the research was conducted in the absence of any commercial or financial relationships that could be construed as a potential conflict of interest.

## Abbreviations

AEDs: anti-epileptic drugs
GABA: gamma aminobutyric acid
EMG: electromyograms
EEG: electroencephalogram
MRI: magnetic resonance imaging
PET-CT: positron emission tomography/computed tomography.
